# Evolution of Health Information Sharing Between Health Care Organizations: Potential of Nonfungible Tokens

**DOI:** 10.2196/42685

**Published:** 2023-04-12

**Authors:** Pouyan Esmaeilzadeh

**Affiliations:** 1 Department of Information Systems and Business Analytics College of Business Florida International University Miami, FL United States

**Keywords:** health information exchange, HIE, personal health information, PHI, blockchain, nonfungible token, NFT, evolution of technology

## Abstract

This study attempts to explain the development and progress of the technology used for sharing health information across health care organizations (such as hospitals and physicians’ offices). First, we describe the strengths and weaknesses of traditional sharing models, health information exchange (HIE), and blockchain-based HIE. Second, the potential use of nonfungible token (NFT) protocols in HIE models is proposed as the next possible move for information-sharing initiatives in health care. In addition to some potential opportunities and distinguishing features (eg, ownability, verifiability, and incentivization), we identify the uncertainty and risks associated with the application of NFTs, such as the lack of a dedicated regulatory framework for legal ownership of digital patient data. This paper is among the first to discuss the potential of NFTs in health care. The use of NFTs in HIE networks could generate a new stream of research for future studies. This study provides practical insights into how the technological foundations of information-sharing efforts in health care have developed and diversified from earlier forms.

## Introduction

People may need to visit different health care providers (such as specialists) in their lives because they may encounter various health issues. Providers need to access accurate and complete patients’ past medical records to make informed treatment decisions and increase the effectiveness and efficiency of care delivery. Accessing limited or incomplete information can cause duplication of health care services, such as laboratory tests and repetition of therapy. In addition, as physicians need to search for missing information, administrative costs increase, which could cause delays in providing care and slow down the providers’ workflow. Thus, it is essential for treating physicians to access, integrate, and share patients’ test results and medical procedure records conducted by various providers. However, health care organizations are not necessarily affiliated and may use different systems and standards for storing patient information (such as diverse electronic health records [EHRs]). Seamless sharing of personal health information (PHI) is a demanding project in a highly fragmented US health care system [1]. Fragmented health care services may also challenge how health care providers exchange health-related data as they may use various exchange mechanisms [2]. Thus, different standards for the data storage model, data documentation process, and data transfer mechanism may be used by health care providers in the United States [3]. Health data sharing is an endeavor involving stakeholders such as data owners, data users, and regulators. Sharing health data among health care entities can yield several benefits that include improving care coordination, care quality, and patient safety while reducing mortality rates, medical errors, and health care costs [4]. The ultimate goal is to enable all health care providers to share accurate, timely, and complete medical data nationwide with other entities regardless of where the patient has been treated previously. To achieve this objective, various exchange mechanisms and sharing systems have been used in health care. These systems have some advantages and drawbacks because of the supporting technology and implementation issues. Technological advancements have been used to address the challenges associated with the preceding ones and create new generations of sharing mechanisms. The following sections explain the 3 existing information-sharing mechanisms in health care and propose a new approach to fundamentally address critical issues in mainstream sharing efforts.

## Ethical Considerations

As no human participants were used, this study was exempted from obtaining approval from an institutional review board.

## Information-Sharing Mechanisms in Health Care

### Traditional Information-Sharing Models

The first sharing method in health care was a paper-based exchange or mail transmission. Gradually, health care providers used other conventional methods (such as phone, fax, or information carried on CDs) to exchange patients’ records. Information flows among disparate health care institutions can still be managed through traditional methods such as fax, paper mailing, and phone calls. However, previous studies have reported serious issues associated with nonelectronic data exchange among providers, such as the inability to provide timely access to patients’ medical records and unnecessary tests [5]. As traditional systems cannot integrate patient data into a central hub, paper-based records such as fax or mail could be lost during the treatment period. Because medical data could be sensitive, losing them can increase privacy risks. It is also inconvenient for patients to carry paper-based records or CDs from one hospital or physician’s office to another. The storage of patient records in paper-based folders or CDs also leads to huge maintenance costs for large hospitals. Moreover, keeping folders may cause numerous security risks, such as the threat of natural events and disasters. Offline-based exchange requires additional work, such as copying paper-based files or storing diagnostic images on a CD or a memory stick. Thus, traditional methods cannot be expected to reduce health care costs. Finally, it is unclear to patients who can access, view, and share their paper-based medical records because no alert, alarm, or security safeguards are available to protect offline patient data from unauthorized access.

### Health Information Exchange

The health care industry is currently transitioning from the offline sharing of patient health information to web-based sharing through electronic health information exchange (HIE). HIE allows for web-based transfer of medical data and patient records among health care providers and institutions, providing access to accurate and up-to-date health information across different health care settings. This enables clinicians to make more informed and effective health care decisions, ultimately improving patient outcomes [6]. The primary objective of creating ecosystems for HIE systems is to promote the secure and efficient sharing of patient data on a national scale in the United States. HIE networks facilitate interoperability between various health care entities, intending to improve the quality of care, optimize clinical workflow, provide timely access to patient records, enhance connections between different organizations, and improve overall health care efficiency [7]. There are 3 main mechanisms of HIE: direct exchange, look-up systems, and patient-centered exchanges. Direct exchange involves authorized and trusted health care providers sharing patient data directly with one another. Look-up systems use a centralized database that enables providers to send query messages and request patient records. Patient-centered HIE gives patients greater control over their health information by allowing them to collect and aggregate data from various providers and share it with other health care entities, as needed. This approach enables patients to be more active in managing their health and ensuring that their health care providers access comprehensive and accurate health information [8].

In many developed countries, HIE programs are key policy areas aimed at improving care coordination by facilitating the sharing of accurate and comprehensive health information across health care providers and organizations. HIE databases can be used for various purposes, such as health care decision-making and clinical research. However, despite the potential benefits of HIE, such as improved coordination, reduced costs, and enhanced patient safety, insufficient participation of clinicians in data exchange networks can lead to incomplete HIE databases and reduce the overall value of HIE. In addition, the use of HIE mechanisms presents several challenges. Privacy concerns and the risk of data breaches are 2 important barriers to electronic data sharing in the United States. These factors must be addressed to ensure that HIE programs can achieve their objectives and realize their full potential for improving health care outcomes [9]. Patients’ concerns about the privacy and security of their information can lead to incomplete medical records in HIE databases because of information-blocking behaviors. Owing to concerns about information integrity and confidentiality, patients may be less likely to participate in data-sharing efforts. This can create a challenge for health care providers who rely on accurate and complete medical records to deliver high-quality care. To address these concerns, it is essential to implement robust privacy and security measures that can help reassure patients and build trust in HIE systems.

In addition, efforts should be made to increase patient awareness and education regarding the benefits of participating in data-sharing programs while ensuring that their privacy and security concerns are adequately addressed [10]. Incomplete information in HIE systems suggests that not all essential data sources from patients are being integrated and accumulated, potentially owing to concerns over privacy and security risks. Even patients concerned about privacy and security may not fully appreciate the benefits of data sharing and its potential impact on public health. Consequently, they may be reluctant to consent to the disclosure of their data to different health care providers. To address this issue, it is crucial to increase patient awareness and education about the potential benefits of HIE systems while ensuring that their privacy and security concerns are adequately addressed. This can help build trust and confidence in the system, which can lead to more complete and accurate data being shared across health care entities [11]. Some health care providers may choose not to participate in HIE networks because of concerns over patient privacy and security and legal implications. These providers may hesitate to share patient health information, which can hinder the success and effectiveness of HIE systems. To address these concerns, it is important to establish clear guidelines and regulations regarding the collection, use, and sharing of patient health information within HIE networks. This can help mitigate legal risks and ensure that patient privacy and security are protected, while enabling the effective sharing of health information across health care entities. In addition, increasing education and awareness among health care providers about HIE systems’ benefits can help build trust and encourage greater participation in these networks [12].

The primary challenges in implementing HIE systems are often attributed to organizational, governance, and technical barriers. These include limited interoperability between different health care information systems, a lack of standardized protocols and procedures for data sharing, and difficulties in coordinating and managing various health care entities and stakeholders involved in the HIE network. Addressing these barriers requires careful planning, collaboration, and investment to develop robust technical infrastructure, governance models, and organizational frameworks that support effective HIE implementation and operation. In addition, the ongoing evaluation and monitoring of HIE systems can help identify and address any ongoing barriers or challenges [13]. The implementation of HIE systems may face several obstacles, including the absence of proper governance structures, insufficient commitment from senior leadership, uncertain return on investment from HIE investment, inadequate technological infrastructure, absence of technical standards for promoting interoperability, challenges in integrating EHR data, insufficient adoption of certified EHRs, compliance with the Health Insurance Portability and Accountability Act (HIPAA) regulations, and insufficient technical training [14].

Furthermore, inadequate collaboration from EHR vendors, limited interorganizational partnerships with other health care entities, apprehensions about patient attrition due to HIE participation, and varying consent policies across different states are further challenges health care organizations may encounter when implementing HIE systems. The literature on HIE highlights that health care providers are worried about losing patients and their associated revenue when sharing data with competing organizations [15]. Competing health care organizations may view information blocking as a way to gain a competitive advantage by controlling patient flow. To maintain their competitive position in the market, these organizations may choose to only partially exchange health information or engage in information blocking altogether. However, framing HIE agreements with partners and reaching data use agreements about HIE can be complex and difficult, which presents a considerable barrier for HIE partners [16].

In the United States, financial incentives and mandates have been provided to encourage the participation of providers and clinicians in HIE projects [17]. An example of this is the federal Meaningful Use program, which outlines the standards for implementing certified EHRs [18], promoting the smooth flow of health information to enhance collaboration and care coordination, and reducing redundant tests and diagnostics, ultimately leading to cost savings in health care [19]. In addition, the Fast Healthcare Interoperability Resources standard is a recent effort to offer adequate beneficial interoperability without the complexity associated with a comprehensive interoperability solution [20]. The advancement of interoperability and transparency in providers’ efforts to achieve this is largely supported by various organizations, such as the Office of the National Coordinator for Health Information Technology within the Department of Health and Human Services, as well as the Centers for Medicare & Medicaid Services and state governments [8]. Despite the policies and incentives provided, widespread participation of providers and clinicians in HIEs cannot be guaranteed. Previous studies have shown that although some hospitals have implemented HIEs, their clinicians may not fully use them to share all types of clinical information with all health care entities, including unaffiliated ones [5].

### Challenges Related to Existing Sharing Models in Health Care

In light of the literature review, the main issues with current exchange mechanisms can be categorized into 4 groups. The first challenge is that mainstream sharing models are mainly centralized and controlled by a health care organization, and they define a minor role for patients in the sharing process [21]. HIE networks mainly focus on EHRs and centralized mechanisms managed by middlemen. The second concern is the low visibility in the security and transparency of sharing mechanisms and a lack of trust in sharing procedures and technologies [22]. The threat of a data breach, a single point of failure, and unclear permission processes are some dimensions of privacy and security issues. The third reason is data quality issues such as outdated data in HIE databases, incomplete or inaccurate patient information stored in HIE databases, and mutability, as any entity participating in HIE initiatives can remove patients’ medical records. The final issue is that the current models do not openly delineate data ownership, and it is not clear who is the owner of the clinical data.

### Blockchain-Based *HIE*

Previous studies suggested blockchain as an alternative to mainstream HIE systems [23-25]. Blockchain is an emerging technology that enables secure and decentralized approaches to reduce the technical risks and governance challenges associated with sharing sensitive health data. Blockchain technology could be a promising platform for sharing information among stakeholders with different interests [26]. Blockchain-based platforms can be a technological solution to foster trustworthy relationships between different business entities (such as affiliated and unaffiliated health care organizations) [27]. Blockchain solutions can improve information management’s authenticity, security, and confidentiality using ledgers, encryption, and distributed networks [28]. By removing intermediaries, blockchain enables data ownership and gives users more control over their data [29].

One practical use of blockchain is to share health information [30]. Previous studies have presented potential advantages of blockchain technology in response to the traditional risks associated with conventional information exchange models [31]. For instance, in the context of HIE, a permissioned blockchain-based network has been suggested as a more secure option to enable the electronic exchange of clinical data with providers [32]. Because medical records are considered sensitive data, legal, consent, and privacy concerns are the top challenges individuals may encounter in sharing their health information [33]. Existing data-sharing models mainly use central data-management mechanisms, making data ownership and controlled access more complicated for individuals. Centralized applications cannot allow multiple stakeholders to actively participate in data-sharing governance. Furthermore, because of nonautomated consent mechanisms and data access management, the custody and administration of data sharing using traditional HIE are complicated [34].

Therefore, decentralized platforms that use encrypted databases are an effective alternative that enables independent stakeholders to supervise data contributions and access [35]. As there is neither a central administration nor a third party, trust will be placed in the network and distributed ledger to collect, store, and validate data sharing among data contributors [36]. Thus, previous studies have offered blockchain-based platforms for health data transmission between patients, providers, hospitals, and research organizations [37]. Decentralized networks of distributed nodes are deemed useful for reducing the inefficiency, costs, trust, and security risks of using central data sets across different boundaries. Data transmission through blockchain platforms can enable data contributors to maintain autonomous and ongoing control of their own data [38]. In these peer-to-peer platforms, each node consists of network participants (such as patients) that collectively contribute to the process of transaction validation and store the same copies of all data-sharing records.

### Types of Blockchain *HIE* Systems

In the context of health care, there are 2 main types of blockchain networks: permissioned and federated [39].

- Permissioned blockchain: a permissioned blockchain is a closed network in which access is restricted to a defined group of participants [40]. Only authorized users can participate in the network and are typically required to pass identity verification checks before they can access the blockchain. Permissioned blockchains are often used in health care to ensure data privacy and security, as they provide a higher level of control over who can access and participate in the network.
- Federated blockchain: a federated blockchain is a network where multiple independent organizations come together to participate in a shared blockchain [41]. Each organization operates its own node on the blockchain, and the nodes work together to validate transactions and maintain the integrity of the network. Federated blockchains are often used in health care to enable information sharing between different organizations, such as hospitals, clinics, and insurance companies, while maintaining some control over who can participate in the network.

The key difference between permissioned and federated blockchains is the level of control over those who can participate in the network. In a permissioned blockchain, access is tightly controlled and only authorized users can participate. In a federated blockchain, there is more flexibility in terms of who can participate; however, the network is still designed to maintain some level of control over the participants to ensure security and data privacy. Both types of blockchains have their own advantages and disadvantages, and the choice of which one to use depends on the specific needs of the health care organization and the use case at hand. For example, a health care organization that is primarily concerned with data privacy and security may choose a permissioned blockchain, whereas an organization that wants to enable information sharing between multiple entities may opt for a federated blockchain.

Several private companies have already offered blockchain-based data-sharing platforms [42]. For example, health care organizations can run their health network on the Ethereum platform to provide different providers with access to treatment information. Caregivers can review the historical interactions between medical experts and patients, which enhances the transparency of the entire medical environment.

### Smart Contracts

Blockchain-HIEs can use smart contracts, programmable computer protocols that verify and execute terms based on predetermined factors. A smart contract is a self-executing contract, with the terms of agreement between the buyer and seller being directly written into lines of code. The code and agreements contained therein exist on a blockchain network, and the contract is automatically executed when certain conditions are met [43]. Smart contracts are typically written in a high-level programming language, such as Solidity for the Ethereum blockchain, and are compiled into bytecodes that can be executed on the blockchain [44]. The code is stored on the blockchain, making it tamper-proof and transparent, and it can be accessed and executed by anyone on the network. One of the key benefits of smart contracts is that they enable trustless transactions, meaning that parties can exchange value without the need for a trusted intermediary. This can reduce transaction costs, increase efficiency, and improve security and transparency. Smart contracts can also be used to automate complex business processes, reduce fraud and errors, and increase accountability [45].

In an HIE setting, smart contracts can be used to automate the sharing and exchange of health data between different entities in the health care ecosystem, such as hospitals, clinics, insurers, and patients. Some examples of how smart contracts can be applied in HIE settings are as follows:

1. Access control: smart contracts can be used to control those who have access to patient health data and under what conditions. For example, a smart contract could be programmed to only allow a patient’s primary care physician to access their medical records or only a researcher to access anonymized data for a specific research study.
2. Consent management: smart contracts can be used to manage patient consent for sharing and using their health data. For example, a smart contract could be programmed to automatically grant or revoke consent based on certain conditions, such as the completion of a clinical trial or expiration of a consent period.
3. Payment management: smart contracts can be used to automate the payment and reimbursement processes for health care services. For example, a smart contract can be programmed to automatically process insurance claims and reimburse health care providers, based on predefined rules and conditions.
4. Compliance monitoring: smart contracts can be used to monitor and enforce compliance with health care regulations and standards. For example, a smart contract could be programmed to automatically verify that a health care provider has met certain quality standards or that a patient’s health data has been handled in compliance with HIPAA regulations [46].

By using smart contracts in an HIE setting, it is possible to streamline and automate many of the processes involved in exchanging and using health data, while also improving data privacy, security, and transparency. Smart contracts can also reduce the administrative burden on health care providers and increase trust among patients and other stakeholders in the health care ecosystem.

## A Proposed Approach: Nonfungible Token Protocols in HIE

### *Nonfungible Tokens:* General Definitions and Examples

In addition to mainstream information-sharing mechanisms, this study also suggests a new approach to HIE efforts. We believe that this new system can leverage the application of nonfungible tokens (NFTs) in HIE networks. Because the concept of NFT is still novel, some basic information is required before NFT-enabled HIE is explained. NFT is generally a new method of digital authentication, as this protocol can be the process or action of proving or showing something genuine or valid. So far, the primary use cases of NFT are in sports moments, collectibles, video games, digital art, music, virtual worlds, fashion, trading cards, and domain names [47]. The NFT protocol is an alternative to the US copyright system, a government body that grants producers (artists) a certificate that can prove the work (artwork) is theirs. Using NFTs, individuals do not need a third party to manage their approval process. Instead, the authentication process can be performed through the Ethereum blockchain as the work (artwork) becomes digital with a certificate (token) [48]. If people download a file (art), this does not mean that they own it. This means that they have a copy of the art that is not original.

When an artwork becomes an NFT, individuals are likely to acquire it because they want to claim ownership of a rare and unique piece of the original art. NFT protocols can also protect artists by enabling one-on-one relationships between them and fans. NFT can help artists sell their products (eg, music and painting) directly to buyers without the involvement of a middleman such as a record label company. There are several reasons why people are eager to accept NFTs instead of copying and pasting artwork. Previous studies have highlighted several reasons why people enjoy purchasing and collecting NFTs [49]. The main motives are uniqueness, greater security than physical collectibles, potential to make money, competitive aspects, entertainment, and connection to an innovative community. Thus, we can define the NFT value based on the following formula: reputation of the creator (eg, artist) + utilities offered (for instance, sending the original tangible artwork) + ownership history (who owned the NFT before and how many times it has been sold) + future value (as a rare digital product).

### The Role of Speculation in the Finances of *NFTs*

Speculation plays a major role in the financial aspect of NFTs. NFTs are unique digital assets that can represent the ownership of a particular item or piece of information, and the perceived rarity and demand of an asset often determine its value. As a result, NFTs have become popular assets for investors and collectors, leading to a surge in speculative buying and selling [50]. One factor driving the speculation in NFTs is the limited supply of certain assets. For example, a rare piece of artwork or a memorable moment in sports can be converted into an NFT, and the scarcity of such assets can increase their value in the market. In addition, the hype around certain NFTs can contribute to speculative buying, as investors seek to capitalize on the perceived value of a particular asset. The speculative nature of NFTs has led to considerable price volatility, with some NFTs selling for millions of dollars, whereas others fail to attract any buyers. This unpredictability can make NFT investment risky because the market can be influenced by various factors, including changing consumer tastes and technological advancements. Despite these risks, many investors and collectors continue to view NFTs as a valuable addition to their portfolios, and the popularity of NFTs is likely to continue to grow as technology and use cases evolve.

### Technical Foundations of *NFT*

#### Metadata

NFTs are a type of digital asset stored on a blockchain, such as Ethereum. NFTs are unique, meaning that each NFT has a distinct value and cannot be replicated or duplicated. However, it is important to note that NFTs themselves do not contain the data in question but rather a very small collection of metadata that provide information about the asset [51]. For example, an NFT representing a digital artwork might include metadata such as the artist’s name, the title of the artwork, and the date of creation. The actual artwork itself would be stored elsewhere, such as on a centralized server or decentralized storage platform such as the InterPlanetary File System [52]. When an NFT is purchased, ownership rights are recorded on the blockchain, making it a transparent and immutable record of ownership. NFT can be transferred to another owner by sending it to a digital wallet address.

#### Copyright

Copyright issues can arise with NFTs because they provide a way to monetize digital assets that may not have been possible previously. This has led to some controversy regarding NFTs and their impact on the art world and other creative industries. One issue is that NFTs do not necessarily confer ownership of the underlying asset but rather a unique identifier that is linked to the asset [53]. This means that someone who purchases an NFT representing a digital artwork may not actually own the copyright to that artwork and may not have the right to reproduce or distribute it without the artist’s permission. Another issue is that NFTs can be used to monetize assets that were previously freely available on the Internet, such as memes or other forms of user-generated content. This has led to concerns that NFTs could be used to profit from the work of others without their consent. Overall, although NFTs offer a new way to monetize digital assets and provide a mechanism for creators to protect their work, they also raise important questions regarding ownership, copyright, and the value of digital art and other assets.

### Potential Application of *NFTs* in *HIE*

This section describes the potential application of NFTs to create digital proof of ownership in HIE. NFTs are recognized as a new way of creating value in various industries; however, they are still in their infancy and are challenged by speculation and inadequate regulations [54]. NFTs, as blockchain-based cryptographic assets that denote proof of ownership for digital objects, can be used in health care to authenticate digital PHI. All test results, treatments, medications, prescriptions, and care plans were considered PHI. NFTs can be produced on permissioned or federated blockchains, which provide a digital token of ownership for PHI. NFTs assigned to PHI can reduce health care organizations’ time and effort to verify critical documentation, thus refining administrative operations of information sharing. NFT-based HIE issuing certificates can eliminate the workload of record keeping, with each medical record having a unique NFT that can be checked for authenticity. Moreover, issuing certificates on the blockchain-enabled HIE makes digital records resistant to tampering, which decreases the chance of encountering fraudulent PHI.

### Network Topology

This section explains the type of blockchain that would be the best network for the proposed NFT-based HIE. Permission-less blockchains are open and decentralized. As no central entity can manage membership or ban illegitimate readers or writers, any individual can join and leave the network as a reader and writer at any time [40]. Thus, the stored on-chain content is readable by all members. However, permissioned blockchains authorize a limited set of readers and writers. Thus, a central entity decides and grants members the right to participate in the write or read operations of the blockchain [55]. Readers and writers can operate in separate parallel interconnected blockchains to promote privacy. To justify the best choice between permission-less and permissioned networks, the properties of these networks suggested by previous studies [56] can be evaluated as follows: (1) public verifiability enables anyone to verify the correctness of the system’s state. For example, each state transition is confirmed by miners in the Bitcoin blockchain; (2) transparency explains the amount of information that should be transparent to an observer and the extent to which every participant can access every piece of information; integrity describes the extent to which health information is protected from unauthorized modifications; (3) redundancy in a blockchain-based HIE is mainly provided through replication across writers; and (4) trust anchor has the highest authority of a blockchain-enabled HIE system to grant and revoke read and write access to the system.

Moreover, to evaluate the best blockchain option for NFT-based HIE, we can use the following evaluation framework:

- Security: the blockchain option should be secure, ensuring the privacy and confidentiality of health information.
- Scalability: the blockchain option should be able to handle a large number of transactions without compromising performance.
- Governance: the blockchain option should have a transparent and robust governance mechanism to ensure the integrity of the data stored on the blockchain.
- Accessibility: the blockchain option should be accessible to all participants in the HIE network.
- Interoperability: the blockchain option should be able to work seamlessly with other existing systems and technologies.

Permission-less blockchains (such as Bitcoin and Ethereum) have a high level of security because they use a distributed ledger system that is difficult to hack. However, they are unsuitable for HIE owing to their limited scalability and governance issues. Permission-less blockchains can handle only a limited number of transactions per second, which is insufficient for large-scale HIE networks. Permission-less blockchains are also unsuitable for handling sensitive health information because of their lack of privacy and confidentiality. In contrast, permissioned blockchains (such as Quorum and Ripple) offer better security and privacy than public blockchains and also provide a good balance between security and scalability. They are scalable and can handle a large number of transactions per second, making them suitable for HIE networks. Permissioned blockchains can provide the required level of governance for HIE networks, as they allow only authorized parties to participate in the network, maintaining the transparency and accessibility of the network. However, permissioned blockchains can be more expensive than public blockchains and may require more resources for maintenance.

On the basis of the evaluation framework, the best blockchain option for an NFT-based HIE is a permissioned blockchain owing to several factors. First, permissioned blockchains offer higher security than public blockchains, because they allow only authorized participants to join the network. This ensures that sensitive health information is protected from unauthorized access or tampering.

Second, permissioned blockchains are scalable and can handle many transactions per second, making them useful in HIE networks. This is particularly important for HIE networks because they require the ability to handle a large volume of transactions while maintaining the integrity of the data. Third, permissioned blockchains provide a transparent and robust governance mechanism that is essential for ensuring the integrity of the data stored in the blockchain. This allows for a higher level of accountability and trust among participants in the network. Fourth, permissioned blockchains offer accessibility to all participants in the HIE network, as they allow authorized users to join the network and access data. This ensures that all relevant stakeholders can access the information they need to make informed decisions. Finally, permissioned blockchains are interoperable, meaning they can work seamlessly with other existing systems and technologies. This is particularly important for HIE networks, as they must integrate various health care systems and technologies to ensure the smooth exchange of health information.

In summary, a permissioned blockchain is the best option for NFT-based HIE owing to its high level of security, scalability, governance, accessibility, and interoperability. By using a permissioned blockchain, stakeholders in the health care industry can ensure secure and efficient exchange of sensitive health information while maintaining transparency and accountability among all participants in the network.

### Consensus Mechanism

The consensus mechanism is a critical aspect of blockchain technology because it enables all nodes in the network to agree on the state of the ledger and improve their fault tolerance [57]. The consensus mechanism determines how new transactions are verified and added to a blockchain. The 2 main types of consensus mechanisms used in blockchain technology are Proof of Work (PoW) and Proof of Stake (PoS). Permission-less blockchains, such as Bitcoin and Ethereum, use PoW as their consensus mechanism. In PoW, miners solve complex mathematical problems to verify transactions and add them to a blockchain. The first miner to solve this problem is rewarded with a newly minted cryptocurrency. PoW is a computationally intensive and energy-consuming process, which makes it less efficient and environmentally friendly than other consensus mechanisms.

In contrast, permissioned blockchains such as Hyperledger Fabric and Corda use PoS or other consensus mechanisms such as Practical Byzantine Fault Tolerance (PBFT) or Raft. In PoS, validators hold a stake in the network, and the probability of being chosen to verify transactions and add them to the blockchain is proportional to the size of their stake. PoS is more energy-efficient than PoW, making it a more suitable consensus mechanism for permissioned blockchains.

Furthermore, PoS consensus mechanisms are often faster and can handle more transactions per second than PoW, making them more suitable for permissioned blockchains that require a high transaction throughput. PBFT and Raft, by contrast, offer a faster consensus mechanism by allowing nodes to reach an agreement through direct communication rather than mining.

In summary, permission-less blockchains rely on PoW as their consensus mechanism, which is computationally intensive and energy-consuming. Permissioned blockchains, by contrast, use more efficient consensus mechanisms, such as PoS, PBFT, or Raft, which are faster, more energy-efficient, and more suitable for high transaction throughput. In a permissioned blockchain, the consensus mechanism is designed to be more efficient, scalable, and suitable for the specific use case of NFT-based HIE. One of the most commonly used consensus mechanisms in this permissioned blockchain could be PoS. In PoS, the validators are incentivized to behave honestly as they stand to lose their stake if they act maliciously. PoS is more energy-efficient than PoW, making it a more suitable consensus mechanism for NFT-based HIE. Because permissioned blockchains have a known set of validators, the consensus mechanism can be optimized for efficiency, throughput, and security. Another advantage of permissioned blockchains is the use of other consensus mechanisms such as PBFT or Raft. These consensus mechanisms use direct communication between nodes to reach a consensus, allowing for faster transaction times and higher transaction throughput.

In NFT-based HIE, permissioned blockchains can be designed to accommodate different types of participants, such as health care providers, insurance companies, and patients, each with their own set of permissions and access levels. This ensures that only authorized participants can access the sensitive health information stored on the blockchain. Thus, the consensus mechanism for permissioned blockchains, such as PoS, PBFT, or Raft, is designed to be more efficient, scalable, and suitable for NFT-based HIE. These consensus mechanisms provide a more energy-efficient and faster alternative to PoW and allow customized permission levels for participants in the network, ensuring that sensitive health information is accessible only to authorized parties.

### Authentication Process via *NFTs*

NFTs enable patients to own their medical records. Thus, health care providers’ new entries (eg, test results) can be first encoded as NFTs and then added to the blockchain. Next, the ownership certification of ownership can be sent to the patient node. This authentication protocol can increase the transparency of medical data ownership and offer new ways to claim or enact ownership. All entities in the blockchain (eg, physicians and insurers) are notified of new data entry, but they cannot access, view, and share records because they do not own them. Another characteristic of NFTs is their verifiability, which is their ability to validate asset ownership. Verifiability proposes the protection of digital assets (such as PHI) against security attacks such as tampering, denial of service, spoofing, and repudiation [58]. When a patient grants permission to a treating physician, a smart contract can share the NFT assigned to patient data with the physician to view records for consent. Sharing the NFT designated as a PHI implies that it confers some rights to the holder (for example, analyzing patient data for finding care planning), but legally, patients will remain the original owner. Therefore, a PHI can be considered a commodity that is useful information transferable between health care providers and patients.

In this system, patient data are represented by an NFT, which contains a small amount of metadata that describes the data and links them to the actual data stored in an external system. Thus, on-chain or off-chain modulation can be implemented. Some metadata on health data transfer (such as sender and recipient addresses and purpose of transfer) could be saved on-chain, and some sensitive data (such as medical records and care planning) could be stored in cloud servers, as cloud computing may play a role in the off-chain storage of health data. Off-chain blockchain systems imply computation or data structurally external to the blockchain network [59]. This explains the communication and interplay between on-chain and off-chain storage, computation, and efforts to evaluate their performance. The main advantages of these blockchain systems are improved scalability, reduced data storage requirements, and enhanced data privacy. These features are well-suited to the needs of the health care industry because of the need to manage various types of medical records, patients, and other health-related data.

The NFT acts as a digital asset that the patient can own and control [60]. When a patient grants permission to a treating physician to access their data, this permission is recorded on the blockchain as a transaction that is validated by the network. A smart contract is then used to manage sharing of the NFT assigned to the patient’s data with the treating physician. The smart contract contains a set of rules and conditions that specify the terms of the patient’s consent and the conditions under which the physician is authorized to access the data. For example, the smart contract might specify that the physician is only authorized to view certain types of data for a specific period or that the physician is required to obtain further consent from the patient before sharing the data with other parties. Once the conditions of the smart contract are met, the NFT assigned to the patient’s data is shared with the treating physician, who can access the actual data stored in the external system. The smart contract records the physician’s access to and use of the data, providing an auditable trail of all data accesses and uses. By using smart contracts in this manner, blockchain-based HIE systems can provide patients with greater control over their health data and enable them to securely share it with authorized parties. Smart contracts also enable patients to set specific conditions and rules for using their data, ensuring that they are only accessed and used per their wishes.

One challenge is that when a patient grants permission to a treating physician to access their data, there may be a need to re-encrypt the data for the physician. This requires a considerable amount of computational effort, bandwidth, and storage, depending on the size of the data and level of encryption used. One approach to address this challenge is to use a hybrid encryption scheme that combines symmetric and asymmetric encryption [61]. In this approach, the patient encrypts their data using a symmetric encryption key, which is then encrypted by the physician’s public key using an asymmetric encryption algorithm. The encrypted data and encrypted symmetric key are stored in an external system. When the physician requests access to the data, a smart contract is triggered, and the patient’s private key is used to decrypt the symmetric key, which is then used to decrypt the data. This process ensures that the data remain encrypted at rest and in transit and can only be decrypted by authorized parties. To reduce the computational effort and bandwidth requirements, the data can be compressed before being encrypted and transmitted to the physician. In addition, advanced encryption algorithms such as homomorphic encryption can be used to perform computations on encrypted data, further reducing the need to decrypt the data and increasing privacy and security. It is worth noting that although re-encrypting data for physicians can be computationally intensive, it is a necessary step to ensure the privacy and security of the patient’s data. Using advanced encryption techniques and optimizing the data transfer process can reduce the computational burden and make the exchange of encrypted health data more feasible in blockchain-based HIE systems.

### Patient Nodes or Wallets

It should be noted that in a blockchain-based HIE system, the “patient node” refers to the part of the network that stores and manages the health data of individual patients. The assumption is not necessarily that patients themselves operate a blockchain node but rather that they have control over their own health data and can grant access to it to authorized parties. The patient node can be operated by various entities, such as health care providers, hospitals, or third-party vendors. In some cases, patients may also be able to operate their own nodes if they have the technical knowledge and resources to do so. However, even if patients do not directly operate a node, they can still benefit from the use of blockchain technology in HIE. For example, blockchain can provide patients with greater control over their health data and enable them to securely share it with health care providers and other stakeholders, as needed. Using a blockchain-based HIE system, patients can also have greater confidence that their data are being protected and used in accordance with their wishes.

In NFT-based HIE, patients can have their own nodes or wallets depending on the design of the blockchain network. However, it is important to note that the level of participation and access to the blockchain network for patients may be limited compared with other participants, such as health care providers or insurance companies. Patients can have their own nodes, which are essentially software clients that allow them to interact with a blockchain network. These nodes can be used to access their health information, verify transactions related to their health records, and grant permission to use their data in research or other applications. However, running a node requires technical expertise and resources, which may not be accessible to all patients. If patients have technical expertise and resources, running their own nodes can give them greater control over their health information and ability to participate more actively in the network. However, this option requires more technical knowledge and resources and may not be accessible to all patients.

An alternative option for patients is to use a wallet, which is a digital tool that allows them to store and manage their NFTs representing their health records. The wallet can be used to authorize access to health records and grant permission for their use in different applications. The use of a wallet is generally easier and more accessible to patients than running a node. Patient wallets are generally more accessible and user-friendly, requiring minimal technical expertise. This option provides patients with a more streamlined and convenient way to manage their health information on the blockchain network. In general, patient wallets may be a more suitable option for most patients with NFT-based HIE as they offer a balance between accessibility and control. Patients can use wallets to manage their health information and authorize access to their data, while retaining some level of control and ownership over their data.

Thus, patients can have their own nodes or wallets in NFT-based HIE, depending on various factors, such as the technical expertise of the patient, desired level of control and access to the network, and design of the blockchain network. Although running a node provides more control and access to the network, using a wallet is a more accessible option for patients who may not have technical expertise or resources to run a node. A well-designed NFT-based HIE should provide patients with a range of options for managing their health information on the blockchain network, ensuring that their data are secure, accessible, and under their control.

### Incentivization

In a blockchain-based HIE system, the main challenge is motivating patients to share their medical records with other nodes. Blockchain technology has been suggested to eliminate the inefficiencies, costs, and risks associated with traditional data sharing in health care. Blockchain can also be used to authenticate genuine content [62]. However, the issue with blockchain-based HIE is finding an appropriate and meaningful incentive mechanism to use the promise of data sharing by relying on a decentralized system for data storage and management. As NFTs are nonfungible, their perceived value depends on their content, characteristics, and purpose of use. We can expect that because PHI is unique and its units are noninterchangeable with one another, it is nonfungible. Thus, patients retain ownership of an NFT assigned to PHI and collect royalties (incentives) from sharing their content. Smart contracts can provide reasonable incentives for sharing NFT-based medical records for different purposes. Smart contract terms and conditions can be set based on 2 primary purposes of HIE: health care and medical research.

The NFT assigned to a patient’s PHI is often shared with other physicians for health care reasons such as receiving professional advice, diagnosis, prescription, treatment options, and care planning. In this case, blockchain-based HIE can reward data owners (patients) using recognition points. Thus, blockchain technology can support building incentives for data owners to share their data in exchange for credits encoded in smart contracts [63]. Credits are integrated into blockchain-based platforms and shared with others in the HIE network. For example, the holders of credits will receive recognition for sharing their health data that could be used to improve health quality, help physicians find customized care, reduce health risk factors, and discover the best health care practices. Receiving more points implies that the patient has been actively engaged in their health care procedures. Even gamification concepts, such as points and leveling systems, can be used to calculate engagement scores and rank patients accordingly compared with their peers in HIE networks.

In the second case, disease foundations and academic institutions may ask data owners (patients) to share the NFT assigned to their PHI for clinical research purposes. Blockchain-based HIE can incentivize patients with digital tokens to encourage them to assist in health discoveries and help drive medical innovation for the greater good of humanity. NFTs enable patients to receive royalties each time their PHI is transferred to a new research project. Thus, terms and conditions defined in smart contacts can calculate incentives and electronically reward data owners with cryptocurrencies to share the NFT of medical data for medical searches. For example, owners of NFTs who share their medical records, lifestyle data, and other health information with scientists through a secure platform are not the subjects of research, but are partners in discovering new treatments. In return, patients who share NFTs assigned to health data will receive coins, which can be exchanged with other cryptocurrencies (such as Bitcoin and Ethereum). As patients share NFTs in the network and the value of NFTs varies, incentives can be calculated based on a mix of recency, variety, and volume of medical data, as well as the frequency of sharing. One copy of NFTs exists in this decentralized platform, and patients can control their inclusion in the network and release their consent to how it is used in research. All health data are deidentified, accumulated, encrypted, and stored in the permissioned blockchain. If patients no longer want to contribute to health research, they can revoke permission and remove their NFT assigned to health data from the platform.

### Challenges to Incentivization

Incentivizing users to share data for financial gain in a decentralized and anonymous environment can create challenges related to data quality. When users are incentivized to share data for financial gain, there is a risk that malicious actors will fabricate data sets to take advantage of the incentives [64]. This can result in the creation of large volumes of low-quality or fraudulent data that can be detrimental to commercial users and scientific research. One way to mitigate these risks is to design incentives to reward users for sharing high-quality data validated through independent sources. For example, rewards could be tied to data that a trusted third party, such as a research institution or a regulatory agency can verify independently.

In addition, incentives could be designed to encourage users to share data relevant to specific research or commercial applications and discourage the sharing of data that are not relevant or of poor quality. Another approach for mitigating data quality issues in a decentralized and anonymous environment is to use data validation algorithms to detect and filter out fraudulent or low-quality data. These algorithms can be designed to analyze patterns and anomalies in data to identify potential sources of fraud or errors. Using these algorithms can reduce the risk of fraudulent data and maintain the overall quality of data sets. Overall, it is important to carefully consider the design of incentives and validation mechanisms when incentivizing users to share data in a decentralized and anonymous environment. Using a combination of trusted third-party validation and sophisticated data analysis techniques can incentivize users to share high-quality data while reducing the risk of fraudulent or low-quality data.

### Key Management System

Ensuring safe custody of patient keys is a critical component of any blockchain-based HIE system. One approach to address this challenge is to use a key management system (KMS) designed to securely store and manage cryptographic keys, including private keys [65]. A KMS can offer a range of features and safeguards to protect private keys, such as encryption, access control, and backup and recovery capabilities. For example, a KMS can encrypt private keys using strong cryptographic algorithms and protect them by restricting access controls that can view or modify them. In addition, a KMS can store backup copies of private keys in secure, off-site locations, which can help prevent key losses owing to hardware failures, natural disasters, or other unforeseen events. In addition to using a KMS, several other measures can be taken to ensure the safe custody of patient keys in a health care environment. For example, patients can be educated about the importance of safeguarding their private keys and providing instructions on how to do so. Health care providers can also implement policies and procedures to help patients manage their keys, such as offering secure storage options or periodically reminding patients to check the status of their keys. Ultimately, the key to ensuring the safe custody of patient keys in a blockchain-based HIE system is to balance security with usability. Although it is important to use strong security measures to protect private keys, it is also important to ensure that patients can easily access and manage their keys without undue burden or complexity.

### Data Ownership and Access Control

It is worth mentioning that there is still debate about patients always being the owners of their health data [66]. In most cases, patients are considered the owners of their health data. However, ownership of health data can be a complex issue and may vary depending on the specific situation and jurisdiction. For example, in some cases, health care providers may own certain portions of a patient’s health data, such as test results or clinical notes. In addition, if a patient has agreed to participate in a research study or clinical trial, ownership of their health data may be transferred to researchers conducting the study. The ownership of health data may change for a variety of reasons. One possible reason is when a patient decides to share their health data with a health care provider or another third party for a specific purpose, such as obtaining a second opinion or participating in a clinical trial. In this case, the patient may transfer ownership of their health data to the health care provider or third party for the duration of the specific purpose.

Another possible reason for a change in health data ownership is when a patient agrees to sell their health data to a third party, such as a pharmaceutical company or research organization. In this case, the patient transfers ownership of their health data to a third party in exchange for compensation. Any transfer of ownership of health data should be performed with informed consent from the patient and in compliance with applicable privacy laws and regulations. In addition, patients should be able to revoke their consent and regain ownership of their health data at any time. Even if we assume that patients own their health data, they can remain the owner but share more than one copy of a given health data set with health care researchers (in exchange for incentives) using the design principles of NFT. Thus, data ownership changes in the context of HIE can facilitate data sharing.

NFTs can be useful in tracking the ownership and provenance of digital health information, but they do not inherently provide privacy or secure access. In addition to ownership, access control is an essential aspect of the exchange of health information. Access control determines who has the permission to view, modify, or share health information. Although an NFT can indicate ownership of a piece of digital health information, it does not automatically provide access control. Access control mechanisms must be in place to ensure that only authorized individuals or entities can access information. Several access forms are needed in NFT-based HIE to ensure proper privacy and security of PHI. These access forms are as follows:

- View access: this is the ability to view health information. View access is necessary for health care providers and patients to access their health records.
- Modify access: this is the ability to modify or update health information. Access modification is necessary for health care providers to update patient records with new information, such as diagnoses, treatments, and medications.
- Share access: the ability to share health information with other health care providers or entities. Share access is necessary for health care providers to share patient records with other providers involved in patient care, such as specialists or hospitals.
- Revoke access: this ability to revoke access to health information. Revoke access is necessary for patients to control access to their health records and to prevent unauthorized access.
- Audit access: this is the ability to audit access to health information. Audit access is necessary to track who has access to health records and monitor for unauthorized access.

These forms of access are crucial in ensuring that PHI is properly secured and only accessed by authorized individuals or entities. Although NFTs can be used to track the ownership of digital health information, access control mechanisms must be implemented to ensure the privacy and security of PHI so that only authorized individuals or entities can access it. Thus, NFT-based HIE with access control mechanisms can potentially help solve ownership issues related to health data. In traditional HIE, ownership of health data can be unclear, with different parties (such as health care providers, patients, and health systems) claiming ownership of different aspects of the data. Using NFTs to track ownership of health data can clarify who owns which pieces of data. NFTs with robust access control can be used to create a clear and transparent record of ownership, which can help prevent disputes over ownership of health data. This can potentially streamline the sharing of health information and make it easier for patients to access their own health records by ensuring the proper use and protection of PHI.

### Data Storage, Security, and Cost

On the basis of on-chain or off-chain modulation, the data can be stored in or off the network. In the on-chain model, an NFT will only hold metadata for the health data, not the health data itself, because health information may be too big to be efficiently saved on chain or they could be very sensitive, which could raise privacy concerns. Blockchain technology, which underpins NFTs, has limitations in terms of scalability, and storing large amounts of data on a blockchain can be expensive and slow down the network. However, NFTs can still be useful for securely tracking and managing health data (such as data related to health data transfer between 2 health care organizations). In the off-chain model, an NFT can hold more sensitive data, such as the patient’s name, medical record number, date of birth, and other relevant health-related information. Thus, patient names and other identifiers are not included in the NFT core data because of privacy concerns. On the basis of this modularity, metadata can be used to link the NFT to the actual health data stored in an external system, such as a centralized database or decentralized storage network.

Therefore, the health data must be stored in an external system. For example, health data could be a centralized system, where a single entity or organization is responsible for operating the data storage, maintaining encryption, and standard techniques for securing sensitive data and bearing costs. The choice of encryption scheme would depend on the system’s specific requirements, such as the level of security required, size of the data, and system performance requirements. Some examples of encryption algorithms to secure health data stored in an external system can be advanced encryption standards, RSA encryption, elliptical curve cryptography, and blowfish [67]. Using an NFT to represent a patient’s health data makes it possible to maintain a secure and tamper-proof record of the data ownership, access, and use. This can improve data privacy and security, reduce the risk of data breaches, and increase trust in the health care system.

The entity that operates the binary data storage depends on the specific implementation of the system. In a centralized system, a single entity or organization may be responsible for operating storage. By contrast, in a decentralized system, storage and cost may be distributed among multiple nodes in a blockchain network. In either case, it is essential to ensure that the entity operating the storage has proper security measures in place to prevent unauthorized access and protect data from cyber threats. Regarding the cost related to storing large binary data, the responsible party depends on the specific implementation of the system. In a centralized system, the entity operating the storage unit is typically responsible for bearing costs. In a decentralized system, the cost may be distributed among multiple nodes in the blockchain network. The nodes that store the data may be incentivized by rewards or other compensations. Ultimately, the responsible parties and the cost structure must be determined based on the specific use case and implementation.

### Will *NFTs* Act as New Standards?

Despite the mentioned flaws of traditional HIE systems, they have been tested and tried, and many adhere to strict regulatory requirements, which is not the case for the novel, blockchain-based HIE. Indeed, health care organizations often use different standards, making information sharing more complex [68]. However, it is also important to note that using NFTs in blockchain-based HIE systems does not necessarily imply the creation of a new standard. Rather, NFTs can be viewed as tools for facilitating information sharing across existing standards and systems. By creating a common mechanism for representing and accessing patient data, NFTs can enable health care organizations to exchange data more easily and efficiently, without necessarily requiring them to adopt a new standard. One of the main advantages of blockchain-based HIE systems is that they are designed to operate in a decentralized and interoperable manner, which means that they can work with various standards and systems. By leveraging the power of blockchain technology and NFTs, health care organizations can create a more unified and standardized approach to data sharing without necessarily forcing them to adopt a single, rigid standard. There may still be challenges associated with integrating different standards and systems, and there will likely be a need for ongoing collaboration and cooperation among health care organizations to ensure that data are exchanged accurately and securely. However, by using NFTs in blockchain-based HIE systems, health care organizations can take an important step toward creating a more efficient and effective health care ecosystem that can better meet the needs of patients and providers alike.

### How Health Data Are Exchanged Using *NFTs:* Steps and Processes

NFTs are unique digital assets that represent ownership of a particular item or piece of information. In health care, NFTs can be used in HIE models to secure information exchange between different health care providers. The process of exchanging information using NFTs in HIE models typically involves the following steps:

1. Creation of NFTs: health care providers create NFTs that represent specific pieces of patient information, such as medical records, test results, or imaging studies.
2. Authentication of NFTs: before exchanging information, NFTs are authenticated to ensure that they represent valid and accurate information. This authentication process can include verifying the identity of the health care provider who created the NFT and checking the integrity of the data represented by the NFT.
3. Transfer of NFTs: once authenticated, NFTs are transferred securely between health care providers using blockchain technology. The blockchain ensures that the transfer of the NFT is immutable and tamper-proof, which helps maintain the privacy and security of patient information.
4. Verification of NFT ownership: when a health care provider receives an NFT, they verify the ownership of the NFT to ensure that they have the right to access the patient information represented by the NFT. This verification process involves checking the digital signature associated with the NFT or consulting a blockchain ledger to confirm ownership of the NFT.
5. Accessing patient information: once ownership of the NFT is verified, the health care provider can access the patient information represented by the NFT. This information can be used to inform patient care and treatment decisions.

Overall, using NFTs in HIE models can help ensure secure and efficient information exchange between health care providers, while protecting patient privacy and data security.

### The Architecture of *NFT*-Based *HIE*

The NFT-based HIE mechanism consists of several key components:

1. Health care providers and patients: health care providers (such as doctors, hospitals, clinics, and pharmacies) create and access EHRs for their patients. Patients can also access their own EHRs and share them with health care providers.
2. EHRs: EHRs are electronic records that contain patient health information, including medical history, diagnoses, treatments, and medications. These records are stored in a secure and decentralized manner using the blockchain technology.
3. NFTs: NFTs are unique digital tokens used to represent ownership of digital assets. In the context of NFT-based HIE, NFTs are used to represent ownership of patient EHRs.
4. Smart contracts: smart contracts are self-executing programs that run on a blockchain. In the context of NFT-based HIE, smart contracts are used to automate the process of sharing patient EHRs. Smart contracts define the rules and conditions for sharing EHRs, and ensure that these rules are followed.
5. Data sharing: when a health care provider requests access to a patient’s EHR, the patient can grant permission by transferring ownership of the NFT representing their EHR to the health care provider’s wallet. The smart contract is then executed and the health care provider can access the patient’s EHR.
6. Audit trail: the blockchain maintains a transparent and immutable audit trail of all EHR transactions, providing a secure and reliable record of who accessed what information and when.

**Figure 1:**
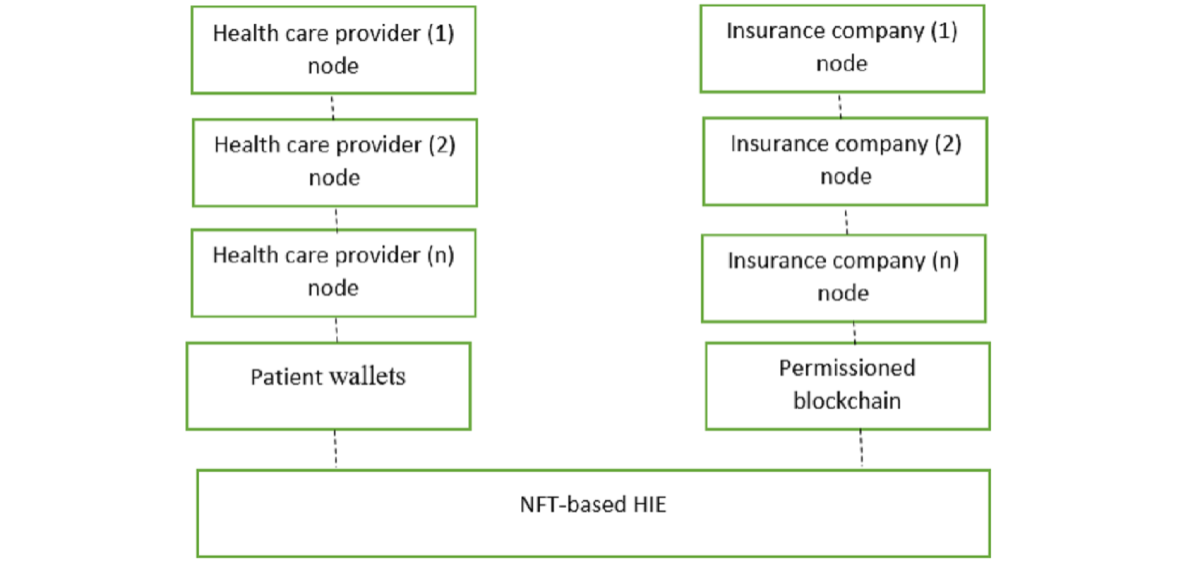
Schematic diagram of relationships between entities in a nonfungible token (NFT)–based network. HIE: health information exchange.

In the first schematic diagram (Figure 1), the NFT-based HIE network comprises various participants, including health care providers, insurance companies, patients, and the blockchain network. Each health care provider has its own node connected to the NFT-based HIE network, enabling it to interact with the blockchain and access patient health records. The insurance company also has its own node connected to the network, enabling it to verify insurance claims and payment transactions. Patients have their own digital wallets or nodes connected to the network, which they can use to manage their health records and grant access to health care providers or insurance companies. When a patient visits a health care provider, the provider creates a new NFT representing the patient’s health record and adds it to the blockchain network. The NFT contains a unique identifier that links it to the patient’s identity and other relevant information, such as the type of medical treatment, date, and health care provider.

The health care provider can then access the patient’s health records through the blockchain network using their own nodes. The insurance company can also verify transactions related to the health care claim and process the payment through its own node that is connected to the network. Thus, this network topology and mechanism enable secure and efficient health information sharing between different NFT-based HIE network participants, while ensuring data privacy, security, and ownership.

**Figure 2:**
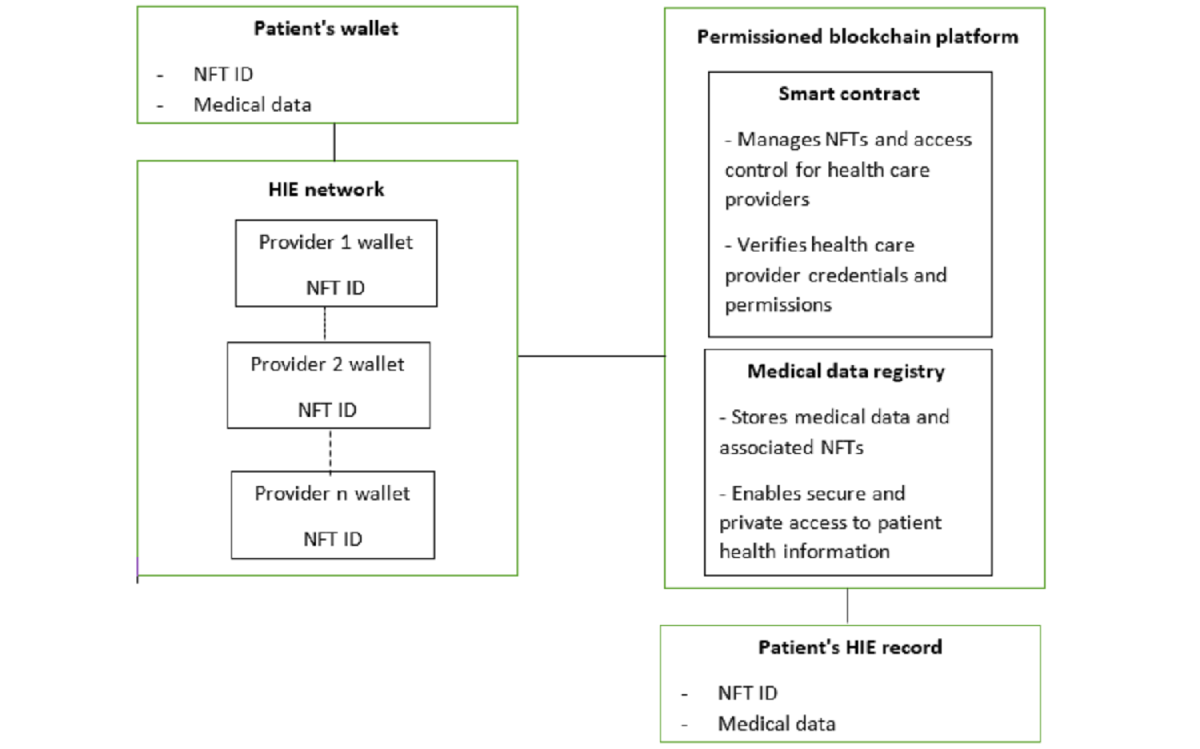
Schematic diagram of how a nonfungible token (NFT)–based health information exchange network works.

The second diagram (Figure 2) schematically shows the operation of an NFT-based HIE network. The patient’s medical data are stored in their wallet as an NFT, containing a unique ID and all their health information. When health care providers need access to this information, they request it from the patient’s wallet through the HIE network. The HIE network uses a smart contract to manage NFTs and access control for health care providers. The provider wallet also contains an NFT that identifies them as health care providers and allows them to access the patient’s health information. Once the provider has verified their identity and permissions, they can access the patient’s health information from the patient’s wallet. The provider can then update the patient’s health information and send the updated data back to the patient’s wallet via the HIE network. All transactions between the patient’s wallet, provider’s wallet, and HIE network are recorded on the blockchain as secure and private transactions. The blockchain also contains a medical data registry, which stores medical data and associated NFTs, and enables secure and private access to patient health information. The patient’s HIE record is a permanent, tamper-proof record of all of their health information. The record is stored as an NFT on the blockchain and is accessible only to authorized health care providers with patient permission. Overall, an NFT-based HIE network provides a secure, private, and decentralized way for patients to control and share their health information, while also ensuring that health care providers have access to accurate and up-to-date medical data.

### Costs and Data Volumes Affecting the Implementation Process

The costs and data volumes can significantly affect the design and implementation of NFTs in blockchain-based HIE systems:

- Costs: the cost of implementing NFTs in blockchain-based HIE systems can vary depending on the system’s complexity, blockchain technology used, and number of participants involved. As NFTs are unique digital assets, the cost of creating and storing them can be high, particularly for large volumes of data. The cost of creating and managing NFTs can also increase as the number of parties involved in the HIE system increases.
- Data volumes: the amount of data being exchanged via NFTs in blockchain-based HIE systems can significantly affect system design and performance. As data volumes increase, the HIE system may need to be designed to handle increased traffic, potentially requiring additional computing power and storage capacity. In addition, as data volumes increase, the system’s security mechanisms must be scalable to ensure that the data are not compromised.

Several strategies can be used to address the challenges posed by costs and data volumes:.

- Optimization of system design: system designers can optimize the design of blockchain-based HIE systems to reduce costs and improve performance. This can include designing the system to scale dynamically, using cost-efficient blockchain technologies, and minimizing the amount of data exchanged via NFTs.
- Data compression and aggregation: to reduce costs associated with NFT creation and storage, data can be compressed and aggregated to reduce the size of the NFT. This can be done by extracting only essential data from patient records, which can help reduce the cost and complexity of creating and managing NFTs.
- Collaborative models: by implementing a collaborative model for HIE, the cost and complexity of managing NFTs can be reduced. In a collaborative model, health care providers can share the costs associated with NFT creation and management, potentially leading to lower costs for all the parties involved.

Thus, cost and data volume considerations must be carefully considered in the design and implementation of NFT-based HIE systems to ensure that the system is efficient, secure, and scalable.

### Concerns About Using *NFTs* in *HIE* Systems

As with any emerging technology, there are criticisms and concerns surrounding the use of NFTs in HIE [69]. Some criticisms include the following:

- Limited scope: although NFTs can potentially transform HIE by providing a secure, decentralized mechanism for exchanging health information, their scope is limited. NFTs can only be used to exchange specific pieces of information, such as medical records or test results. They cannot be used to exchange real-time data, such as patient vitals, which are critical for health care decision-making.
- Lack of interoperability: one of the key challenges in HIE is interoperability—the ability of different systems to exchange and use information. Although NFTs can provide a secure mechanism for exchanging information, they may not be interoperable with existing HIE systems. This can limit its usefulness and adoption.
- Regulatory challenges: the use of NFTs in HIE raises regulatory challenges, including issues related to data privacy, security, and ownership [69]. The regulatory landscape for NFT-based HIE is still evolving, and it is unclear how regulators approach these challenges.
- Technical challenges: the technical challenges associated with designing and implementing NFT-based HIE systems can be noteworthy. These challenges include ensuring the scalability and performance of the system, managing the costs associated with NFT creation and management, and ensuring the security of the patient data.

Thus, although NFTs can potentially transform HIE, some challenges and limitations need to be addressed. To effectively integrate NFTs into HIE, careful consideration must be given to these challenges and potential solutions must be explored.

### Challenges and Suggestions for Future Studies

The concept of NFTs is suggested to address a long-standing problem related to the proof of ownership for PHI by offering a mechanism to validate who could own the medical data in the HIE networks. However, our study is among the first attempts to highlight this opportunity, and it is far from achieving this goal, with several questions remaining regarding the legal, financial, and user aspects. The first challenge regarding the application of NFTs in HIE projects can be viewed from the perspective of regulatory considerations. Topics related to NFTs are still novel; thus, a lack of regulation may facilitate fraudulent activities and increase uncertainty regarding the use of NFTs in health care. As the NFT sector is currently prone to fraud, such as phishing activity in the digital asset domain, new dedicated regulations are required to distinguish the application of NFTs in health care. For example, a new amendment to HIPAA is required to articulate how a blockchain-based HIE in which NFT protocols are embedded can be used nationwide.

Moreover, in the United States, different states have diverse rules and regulations regarding the ownership of medical data (ranging from no clear laws to stringent regulatory frameworks). Because of various regulatory strictness, some states will likely create favorable environments that try to adopt applications of NFTs in HIE networks; other states might ban the use of NFTs outright. It would be an interesting research area for future studies to shed more light on the concept of NFTs (especially in health care) from a regulatory perspective.

The second challenge is the cost of creating NFTs. A possible barrier is the additional cost of minting NFTs. In this case, how would this impact cost and convenience, and who will bear the cost of creating and minting associated NFTs? For instance, do care providers and patients jointly contribute to creating patients’ medical records, or is this responsibility for health care organizations? These questions can explain the complexity of adding NFTs to the blockchain HIE and the incremental benefit of this change. Thus, there is a lack of clarity on whether expanding NFTs’ functions in health care is a financially feasible project. Minting NFTs assigned to PHI on a permissioned blockchain requires a robust technological infrastructure with stringent security safeguards. Therefore, further research is required to examine the phenomenon of NFTs and their application in HIE from a financial perspective.

Third, NFTs in health care could be promising; however, their implementation remains challenging. Different stakeholders in the health care ecosystem and layers in the NFT-based HIEs architecture require robust protocols for stakeholder collaboration and interaction. For example, most patient visits are attributed to older patients (older than 50 years of age), who may not be technology proficient, may require extensive training to understand the technology, and may need to provide access to providers for their medical records. What would happen if certain medical records (older or generated through nonparticipating providers) cannot be converted to NFTs? What will happen if a patient is incapacitated (or not in the correct mental state) and cannot grant access to medical records for urgently needed care?

The fourth challenge refers to user perception, as little is known about whether potential users of information-sharing projects in health care will accept NFT-based HIE. As the NFT concept is still new and there is a lack of public awareness about this phenomenon, many questions remain unanswered regarding the perceived viability, utility, and value of NFTs. Thus, further studies are needed to investigate how users (such as physicians and patients) may adapt to NFT technology in health care settings. For instance, researchers can examine the value of NFT-based HIE from user perspectives, such as ease of use, usefulness, cost-effectiveness, error reduction, and productivity.

### A Framework for Further Investigation

As the use of NFTs in blockchain-based HIE systems continues to evolve, further research is needed on the design and implementation of these systems. This research could entail the following areas:

- Technical design considerations: there is a need for further research into the technical design considerations of integrating NFTs into blockchain-based HIE systems. This could include exploring optimal blockchain technology for HIE, designing smart contracts that govern NFT exchange and storage, and developing efficient authentication mechanisms.
- Regulatory and legal considerations: there is a need for further research into the regulatory and legal considerations of NFTs in health care. This could include exploring the legal implications of exchanging health care information via NFTs, the potential impact on patient privacy, and the role of regulatory bodies in overseeing the use of NFTs in health care.
- User acceptance and adoption: further research is needed to understand user acceptance and adoption of NFTs in health care. This could involve assessing the usability of NFT-based HIE systems, identifying barriers to adoption, and understanding the perspectives of health care providers and patients.
- Data security and privacy: there is a need for further research into the data security and privacy implications of using NFTs in health care. This could involve exploring the potential vulnerabilities of NFT-based HIE systems, designing robust security mechanisms, and identifying potential threats to patient-data privacy.

Overall, future studies should provide insights into the design and implementation of NFT-based HIE systems that are secure, efficient, and user-friendly while also addressing regulatory and legal challenges and protecting patient data privacy.

## Evolution of Information-Sharing Technology in Health Care

Table 1 summarizes the growth paths of technologies used in information-sharing initiatives in health care. The types of technology and examples of sharing mechanisms for each initiative are described. Moreover, the key challenges of each technology are highlighted, accompanied by the changes required to address those issues, which lead to the transition to the next technological advancement.

**Table 1:** Comparison between different information-sharing initiatives in health care.

Information-sharing initiatives	Type of technology	Example of sharing mechanisms	Challenges	Changes required
Traditional models	Conventional models (paper-based or voice-based)	Fax, mail, CD, and phone calls	The inability to provide timely access to patients’ medical records Performing unnecessary and repetitive medical tests Cannot integrate patient data into a central hub Chance of losing data Inconvenient for patients to carry nonelectronic records Space and costs of storing files Security and privacy risks Additional workload for physicians	Digitalization of medical records Developing a central database to store patient data Facilitating interoperability across multiple health care entities Standards of data storage and transfer should be determined
HIE^a^ networks	Centralized platforms (databases + emails + patient portals)	Direct exchange, look-up, and patient-centered	Implementation issues (organizational, financial, and governance barriers) Lack of certified EHRs^b^ Interorganizational partnerships with unaffiliated health care organizations Trust-based networks Privacy concerns and risks of a data breach Various patient consent policies Lack of transparency on sharing procedures	Developing decentralized networks so that multiple stakeholders can overlook data sharing More transparency in sharing patient data The threat of a single point of failure should be solved More stringent security measures should be applied Data ownership should be clear Better mechanisms for authentication and granting permission to access data should be used
Blockchain-based HIE	Decentralized platforms	Permissioned blockchain, federated blockchain, and smart contracts	Lack of awareness about blockchain applications in health care Lack of regulations and guidelines Little is known about the perceptions of potential users Lack of incentives for sharing medical records	More organizational training and marketing strategies to promote blockchain applications in health care Need for federal and state-based regulations dedicated to the use of blockchain in health care projects Incentive mechanisms are required to encourage information sharing Patient medical data can be treated as a nonfungible asset
NFT^c^-based HIE	Decentralized platforms	Permissioned blockchain, federated blockchain, smart contracts, and NFTs	NFT technology is still novel Lack of dedicated regulations for NFTs Lack of research on the feasibility of NFT-based HIE Market traction	More research is required on the practicality, viability, value, and utility of using NFT technology in health care Types of incentives should be studied New amendments, compliance, and dedicated regulatory framework for NFTs Implementation barriers to minting NFT in health care and required protocols for interactions with stakeholders should be addressed

^a^HIE: health information exchange.

^b^EHR: electronic health record.

^c^NFT: nonfungible token.

## Conclusions

### Overview

This study sheds light on the characteristics of emerging technologies that support health information–sharing efforts. Rapid technological advancements are accompanied by higher security risks, such as authenticity. We evaluated the potential of NFTs as a novel technology that can be leveraged in new use cases such as health care to mainly solve ownership and authenticity problems. The use of NFTs in HIE systems has the potential to revolutionize the health care industry by enabling the secure and efficient sharing of patient health information. NFT-based HIE may perform existing information exchange functions differently. The benefits of using NFTs include enhanced data security and privacy, improved interoperability, and streamlined data exchanges. We believe NFT technology can be a good fit for HIE networks because, first, NFTs are noninterchangeable. Each NFT is linked to a digital PHI that specifies the medical record’s values, ownership, and sharing rights. Second, NFTs are immutable; thus, they cannot be altered, manipulated, or forged in the information-sharing process. Third, every NFT needs to have an owner, and this is a public record that is easy for anyone to verify. In the proposed NFT-based HIE, patients are the original owners of their PHI, and other entities (such as providers) may be granted the right to check, analyze, and share such medical records based on the terms and conditions defined in a smart contract. NFTs can provide secure records of ownership and authentication in HIE networks. However, several challenges must be addressed before the widespread adoption of NFTs in HIE systems. In addition to the distinguishing features of NFTs, this technology presently faces a lack of dedicated NFT regulation due to its novelty and weakly enforced markets. For example, developing a regulatory framework to control NFT activities could help reduce the high degree of uncertainty in NFTs by forcing creators to obey specific guidelines. The level of regulatory clarity regarding NFTs can encourage more entrepreneurs to invest in different use cases (such as in health care). These challenges include the need for technical standards and infrastructure, legal and regulatory issues, and concerns regarding scalability and sustainability. Overall, although challenges need to be addressed, the benefits of using NFTs in HIE systems outweigh their drawbacks and offer promising opportunities for improving health care outcomes. Further research and development are necessary to address these challenges and fully realize the potential of NFTs in HIE systems. This study suggests that adding NFTs to HIE frameworks could be promising; however, further research is required to validate the value of this change.

## Abbreviations

EHR: electronic health record
HIE: health information exchange
HIPAA: Health Insurance Portability and Accountability Act
KMS: key management system
NFT: nonfungible token
PBFT: Practical Byzantine Fault Tolerance
PHI: personal health information
PoS: Proof of Stake
PoW: Proof of Work

## Availability of Data and Materials

All the data analyzed in this study are included in this published article.

## References

[1] Cherry JC, Dryden K, Kobb R, Hilsen P, Nedd N (2003). Opening a window of opportunity through technology and coordination: a multisite case study. Telemed J E Health.

[2] Esmaeilzadeh P (2020). Patients' perceptions of different information exchange mechanisms: an exploratory study in the United States. Methods Inf Med.

[3] Hatef E, Weiner JP, Kharrazi H (2019). A public health perspective on using electronic health records to address social determinants of health: the potential for a national system of local community health records in the United States. Int J Med Inform.

[4] Yeung T (2019). Local health department adoption of electronic health records and health information exchanges and its impact on population health. Int J Med Inform.

[5] Esmaeilzadeh P, Sambasivan M (2016). Health information exchange (HIE): a literature review, assimilation pattern and a proposed classification for a new policy approach. J Biomed Inform.

[6] Vest JR, Gamm LD (2010). Health information exchange: persistent challenges and new strategies. J Am Med Inform Assoc.

[7] Menachemi N, Rahurkar S, Harle CA, Vest JR (2018). The benefits of health information exchange: an updated systematic review. J Am Med Inform Assoc.

[8] Esmaeilzadeh P, Mirzaei T (2018). Comparison of consumers' perspectives on different health information exchange (HIE) mechanisms: an experimental study. Int J Med Inform.

[9] Wright A, Soran C, Jenter CA, Volk LA, Bates DW, Simon SR (2010). Physician attitudes toward health information exchange: results of a statewide survey. J Am Med Inform Assoc.

[10] Yeager VA, Walker D, Cole E, Mora AM, Diana ML (2014). Factors related to health information exchange participation and use. J Med Syst.

[11] Esmaeilzadeh P, Sambasivan M (2017). Patients' support for health information exchange: a literature review and classification of key factors. BMC Med Inform Decis Mak.

[12] Thorn SA, Carter MA, Bailey JE (2014). Emergency physicians' perspectives on their use of health information exchange. Ann Emerg Med.

[13] Feldman SS, Schooley BL, Bhavsar GP (2014). Health information exchange implementation: lessons learned and critical success factors from a case study. JMIR Med Inform.

[14] Wu YH, Cristancho-Lacroix V, Fassert C, Faucounau V, de Rotrou J, Rigaud AS (2016). The attitudes and perceptions of older adults with mild cognitive impairment toward an assistive robot. J Appl Gerontol.

[15] Adler-Milstein J, Pfeifer E (2017). Information blocking: is it occurring and what policy strategies can address it?. Milbank Q.

[16] Grossman JM, Kushner KL, November EA (2008). Creating sustainable local health information exchanges: can barriers to stakeholder participation be overcome?. Res Brief.

[17] Williams C, Mostashari F, Mertz K, Hogin E, Atwal P (2012). From the office of the national coordinator: the strategy for advancing the exchange of health information. Health Aff (Millwood).

[18] Roehrs A, da Costa CA, da Rosa Righi R, da Silva VF, Goldim JR, Schmidt DC (2019). Analyzing the performance of a blockchain-based personal health record implementation. J Biomed Inform.

[19] Blumenthal D, Tavenner M (2010). The "meaningful use" regulation for electronic health records. N Engl J Med.

[20] Braunstein ML (2018). Healthcare in the age of interoperability: the promise of fast healthcare interoperability resources. IEEE Pulse.

[21] Christodoulou K, Christodoulou P, Zinonos Z, Carayannis EG, Chatzichristofis SA (2020). Health information exchange with blockchain amid COVID-19-like pandemics. Proceedings of the 16th International Conference on Distributed Computing in Sensor Systems.

[22] Shen N, Bernier T, Sequeira L, Strauss J, Silver MP, Carter-Langford A, Wiljer D (2019). Understanding the patient privacy perspective on health information exchange: a systematic review. Int J Med Inform.

[23] Esmaeilzadeh P (2022). Benefits and concerns associated with blockchain-based health information exchange (HIE): a qualitative study from physicians' perspectives. BMC Med Inform Decis Mak.

[24] Lee D, Song M (2021). MEXchange: a privacy-preserving blockchain-based framework for health information exchange using ring signature and stealth address. IEEE Access.

[25] Jiang S, Cao J, Wu H, Yang Y, Ma M, He J (2018). BlocHIE: a BLOCkchain-based platform for healthcare information exchange. Proceedings of the 2018 International Conference on Smart Computing.

[26] Abbas Y, Martinetti A, Moerman JJ, Hamberg T, van Dongen LA (2020). Do you have confidence in how your rolling stock has been maintained? A blockchain-led knowledge-sharing platform for building trust between stakeholders. Int J Inf Manage.

[27] Wamba SF, Queiroz MM (2020). Blockchain in the operations and supply chain management: benefits, challenges and future research opportunities. Int J Inf Manage.

[28] Lu Y (2018). Blockchain and the related issues: a review of current research topics. J Manag Anal.

[29] Iansiti M, Lakhani KR (2017). The truth about blockchain. Harvard Business Review.

[30] Xia Q, Sifah EB, Smahi A, Amofa S, Zhang X (2017). BBDS: blockchain-based data sharing for electronic medical records in cloud environments. Information.

[31] Kuo TT, Kim HE, Ohno-Machado L (2017). Blockchain distributed ledger technologies for biomedical and health care applications. J Am Med Inform Assoc.

[32] Esmaeilzadeh P, Mirzaei T (2019). The potential of blockchain technology for health information exchange: experimental study from patients' perspectives. J Med Internet Res.

[33] Soni H, Grando A, Murcko A, Diaz S, Mukundan M, Idouraine N, Karway G, Todd M, Chern D, Dye C, Whitfield MJ (2020). State of the art and a mixed-method personalized approach to assess patient perceptions on medical record sharing and sensitivity. J Biomed Inform.

[34] Nembaware V, Johnston K, Diallo AA, Kotze MJ, Matimba A, Moodley K, Tangwa GB, Torrorey-Sawe R, Tiffin N (2019). A framework for tiered informed consent for health genomic research in Africa. Nat Genet.

[35] Toh S, Pratt N, Klungel O, Gagne JJ, Platt RW, Strom BL, Kimmel SE, Hennessy S (2019). Distributed networks of databases analyzed using common protocols and/or common data models. Pharmacoepidemiology. 6th edition.

[36] Yue X, Wang H, Jin D, Li M, Jiang W (2016). Healthcare data gateways: found healthcare intelligence on blockchain with novel privacy risk control. J Med Syst.

[37] Azaria A, Ekblaw A, Vieira T, Lippman A (2016). MedRec: using blockchain for medical data access and permission management. Proceedings of the 2nd International Conference on Open and Big Data.

[38] Philippakis AA, Azzariti DR, Beltran S, Brookes AJ, Brownstein CA, Brudno M, Brunner HG, Buske OJ, Carey K, Doll C, Dumitriu S, Dyke SO, den Dunnen JT, Firth HV, Gibbs RA, Girdea M, Gonzalez M, Haendel MA, Hamosh A, Holm IA, Huang L, Hurles ME, Hutton B, Krier JB, Misyura A, Mungall CJ, Paschall J, Paten B, Robinson PN, Schiettecatte F, Sobreira NL, Swaminathan GJ, Taschner PE, Terry SF, Washington NL, Züchner S, Boycott KM, Rehm HL (2015). The Matchmaker Exchange: a platform for rare disease gene discovery. Hum Mutat.

[39] Lu Y, Huang X, Zhang K, Maharjan S, Zhang Y (2021). Communication-efficient federated learning and permissioned blockchain for digital twin edge networks. IEEE Internet Things J.

[40] Helliar CV, Crawford L, Rocca L, Teodori C, Veneziani M (2020). Permissionless and permissioned blockchain diffusion. Int J Inf Manage.

[41] Singh S, Rathore S, Alfarraj O, Tolba A, Yoon B (2022). A framework for privacy-preservation of IoT healthcare data using federated learning and blockchain technology. Future Gener Comput Syst.

[42] Olfson M, Wall MM, Blanco C (2017). Incentivizing data sharing and collaboration in medical research-the S-index. JAMA Psychiatry.

[43] Mohanta BK, Panda SS, Jena D (2018). An overview of smart contract and use cases in blockchain technology. Proceedings of the 9th International Conference on Computing, Communication and Networking Technologies.

[44] Hewa T, Ylianttila M, Liyanage M (2021). Survey on blockchain based smart contracts: applications, opportunities and challenges. J Netw Comput Appl.

[45] Wu H, Zhong B, Li H, Guo J, Wang Y (2021). On-site construction quality inspection using blockchain and smart contracts. J Manage Eng.

[46] Lee JS, Chew CJ, Liu JY, Chen YC, Tsai KY (2022). Medical blockchain: data sharing and privacy preserving of EHR based on smart contract. J Inf Secur Appl.

[47] Rehman W, Zainab H, Imran J, Bawany NZ (2021). NFTs: applications and challenges. Proceedings of the 22nd International Arab Conference on Information Technology.

[48] Wilson KB, Karg A, Ghaderi H (2022). Prospecting non-fungible tokens in the digital economy: stakeholders and ecosystem, risk and opportunity. Bus Horiz.

[49] Chohan R, Paschen J (2022). NFT marketing: how marketers can use nonfungible tokens in their campaigns. Bus Horiz.

[50] Belk R, Humayun M, Brouard M (2022). Money, possessions, and ownership in the metaverse: NFTs, cryptocurrencies, Web3 and wild markets. J Bus Res.

[51] Musamih A, Salah K, Jayaraman R, Yaqoob I, Puthal D, Ellahham S (2022). NFTs in healthcare: vision, opportunities, and challenges. IEEE Consum Electron Mag (forthcoming).

[52] Psaras Y, Gipp B, Schubotz M, Scott W, Castro I, Tyson G, Raman A, Trautwein D (2022). Design and evaluation of IPFS: a storage layer for the decentralized web. Proceedings of the ACM SIGCOMM 2022 Conference.

[53] Guadamuz A (2021). The treachery of images: non-fungible tokens and copyright. J Intellect Prop Law Pract.

[54] Chalmers D, Fisch C, Matthews R, Quinn W, Recker J (2022). Beyond the bubble: will NFTs and digital proof of ownership empower creative industry entrepreneurs?. J Bus Ventur Insights.

[55] Peng L, Feng W, Yan Z, Li Y, Zhou X, Shimizu S (2021). Privacy preservation in permissionless blockchain: a survey. Digit Commun Netw.

[56] Wüst K, Gervais A (2018). Do you need a blockchain?. Proceedings of the 2018 Crypto Valley Conference on Blockchain Technology.

[57] Platt M, McBurney P (2023). Sybil in the haystack: a comprehensive review of blockchain consensus mechanisms in search of strong sybil attack resistance. Algorithms.

[58] Wang Q, Li R, Wang Q, Chen S Non-fungible token (NFT): overview, evaluation, opportunities and challenges. aiXiv..

[59] Miyachi K, Mackey TK (2021). hOCBS: a privacy-preserving blockchain framework for healthcare data leveraging an on-chain and off-chain system design. Inf Process Manag.

[60] Blaney JE, Middleton KE (2022). Using NFTs to store health data: a new era or a privacy disaster. Bus Law Int.

[61] Zhang Q (2021). An overview and analysis of hybrid encryption: the combination of symmetric encryption and asymmetric encryption. Proceedings of the 2nd International Conference on Computing and Data Science.

[62] Kietzmann J, Lee LW, McCarthy IP, Kietzmann TC (2020). Deepfakes: trick or treat?. Bus Horiz.

[63] Gunawan IK, Sukmana HT, Ardianto AY, Henderi (2021). Blockchain technology as a media for sharing information that generates user access rights and incentives. Blockchain Front Technol.

[64] Riesco R, Larriva-Novo X, Villagra VA (2020). Cybersecurity threat intelligence knowledge exchange based on blockchain: proposal of a new incentive model based on blockchain and smart contracts to foster the cyber threat and risk intelligence exchange of information. Telecommun Syst.

[65] Khalil U, Malik OA, Uddin M, Chen CL (2022). A comparative analysis on blockchain versus centralized authentication architectures for IoT-enabled smart devices in smart cities: a comprehensive review, recent advances, and future research directions. Sensors (Basel).

[66] Liddell K, Simon DA, Lucassen A (2021). Patient data ownership: who owns your health?. J Law Biosci.

[67] Rehman S, Talat Bajwa N, Shah MA, Aseeri AO, Anjum A (2021). Hybrid AES-ECC model for the security of data over cloud storage. Electronics.

[68] Holmgren AJ, Adler-Milstein J (2017). Health information exchange in US hospitals: the current landscape and a path to improved information sharing. J Hosp Med.

[69] Kostick-Quenet K, Mandl KD, Minssen T, Cohen IG, Gasser U, Kohane I, McGuire AL (2022). How NFTs could transform health information exchange. Science.

